# Study on the Road Performance of Terminal Carboxylated Nitrile Rubber-Modified Epoxy Asphalt Permeable Concrete

**DOI:** 10.3390/ma18081691

**Published:** 2025-04-08

**Authors:** Wei Shan, Shenru Zhang

**Affiliations:** Institute of Cold Regions Science and Engineering, Northeast Forestry University, Harbin 150040, China; shenru_zhang@nefu.edu.cn

**Keywords:** terminal carboxylated nitrile rubber, permeable pavement, epoxy asphalt, overlay effect

## Abstract

In cold regions, the overlay effect often leads to pavement and subgrade distresses, severely compromising the functionality of roads and infrastructure. To address this issue, this study proposes a solution involving permeable pavements and roadbed structures. However, the application of permeable pavement materials in cold regions remains a significant challenge. Building on previous research, this paper introduces a novel pavement material with exceptional mechanical and temperature performance: terminal carboxylated nitrile rubber-modified epoxy asphalt. Specifically, the mechanical properties, viscosity, high-temperature rutting resistance, low-temperature cracking resistance, and modification mechanisms of five terminal carboxylated nitrile rubber-modified epoxy asphalt mixtures with varying terminal carboxylated nitrile rubber contents were investigated. Additionally, the high-temperature, low-temperature, and water stability properties of three types of porous asphalt concrete were compared. The results demonstrate that the incorporation of terminal carboxylated nitrile rubber significantly enhances the mechanical properties and low-temperature cracking resistance of the asphalt without altering the curing time. Although the high-temperature rutting resistance of the asphalt itself decreases, the high-temperature, low-temperature, and water stability properties of the porous asphalt concrete are improved. This improvement is attributed to the chemical reaction between terminal carboxylated nitrile rubber and epoxy resin, which generates a prepolymer containing new substances and forms a stable sea–island structure. This structure promotes a more homogeneous distribution of the asphalt matrix, thereby increasing the cohesive strength and toughness of the asphalt.

## 1. Introduction

The severity of climate change is driving transformations in infrastructure due to the thermal stresses and moisture distribution effects that permafrost changes impose on various infrastructures, including roads [1]. In the cold northern part of China, every year, asphalt roads are damaged due to their special geography and weather temperatures, which in turn incur significant maintenance and resurfacing costs. In the north, the annual operating temperature of roads ranges from −40 °C to 75 °C, and the huge temperature difference of over 110 °C is a great test for asphalt pavement performance [2]. Asphalt pavement not only has to work normally under the coupling of traffic load and temperature load but also has to face the settlement damage of the road triggered by the freezing and thawing of the frozen soil under the road. In the low-temperature environment, there exists a phenomenon: under the influence of temperature, moisture, and the overlay layer, moisture gathers underneath the pavement (overlay layer) along the direction of the temperature gradient [3], and this part of the moisture causes large damage to the pavement after the occurrence of freezing and thawing. The asphalt pavement commonly used in the project is airtight and impermeable, precisely as an overlay to gather this part of the moisture, which is harmful to the project. Secondly, black asphalt pavement also absorbs a large amount of heat [4], which affects the stability of frozen soil in the roadbed, thus inducing uneven settlement of the road.

The overlay effect appears not only on highways but also on high-speed railways [5] and airports, for which the management methods [6] are different. Given the previous methods, this paper proposes for the first time a new management concept, which applies permeable pavement to the management of the overlay effect through the porous structure of permeable asphalt mixture as a water vapor channel so the rising water vapor in the road will be discharged, which theoretically solves the engineering hazards of the water accumulating under the overlay layer. This is a brand-new treatment concept, and it also means facing new challenges.

This study presents a novel solution for the feasibility of managing the overlay effect in cold regions through the application of permeable pavement. It is anticipated that future research will focus on optimizing the overall roadbed and pavement structure by increasing the void spaces within the road structure to reduce water retention, thereby establishing a comprehensive permeable system tailored for cold regions. However, the inherent porosity of fully permeable pavements allows water to permeate the entire road structure directly into the subgrade, which may increase the moisture content of the subgrade and reduce its bearing capacity. More critically, as temperatures drop, the accumulated water in the subgrade can freeze, leading to frost heave and significant pavement damage. To address this, our research team has previously investigated the use of large-sized crushed stone [7] for treating the subgrade section, which facilitates the diversion of water underground, thereby minimizing the migration of moisture from the granular layer during winter and reducing the impact and damage caused by water in cold regions.

Nevertheless, due to the porous nature of permeable pavements, there is a pressing need for asphalt materials with higher strength and cold resistance for the surface layer. This study underscores the importance of developing such materials to enhance the durability and performance of permeable pavements in cold climates. General matrix asphalt makes it difficult to meet the engineering requirements of cold conditions, and the necessary types of modified asphalt are very broad. The most common is SBS-modified asphalt; its excellent low-temperature performance allows it to meet most of the cold regions of the working environment [8]. SBS is similar to the SBR [9,10] and also has a wider range of applications. Crumb rubber in cold regions of asphalt modification also plays a greater role by improving fracture toughness and tensile strength to improve the low-temperature cracking performance of asphalt pavement [11]; polyethylene particles or fibers in the asphalt mixture play a role in the “bridge” to block the expansion of the cracks, which improves the low-temperature performance of asphalt binder [12]. Fibers improve the low-temperature performance of asphalt mixtures, and the fiber network enhances the integrity of asphalt mixtures, delaying the extension of microcracks and thus enhancing the low-temperature performance of the mixture [13,14]. However, the above materials have improved the low temperature performance of asphalt but lack sufficient working strength when utilized in porous permeable asphalt pavements.

The epoxy groups in epoxy resin (EP) can react with the catalyzed formation of polymers or some corresponding curing agents to form a three-dimensional, dense crosslinked network, which greatly enhances its strength durability corrosion and chemical resistance. Epoxy asphalt, as a kind of high-strength concrete, was first used in bridge deck paving for steel bridges and is also increasingly being used in the field of pavements, including epoxy asphalt, epoxy emulsified asphalt, and epoxy concrete [15]. The high strength and high bonding of epoxy resin can precisely make up for the shortcomings of porous asphalt concrete’s lack of structural strength [16]. Compared with the traditional high-viscosity porous asphalt concrete [17], the high-temperature performance, dynamic modulus, fatigue resistance, resistance to freezing and thawing, and hydrothermal resistance have a better performance, but the low-temperature performance of its asphalt is lower than that of high-viscosity asphalt [18]. This is due to the low flexibility of the epoxy resin itself and the problem of hard brittleness after curing at low temperatures, which limits the application of epoxy asphalt in cold environments [19]. Terminated carboxylated nitrile butadiene rubber (CTBN) is one of the ways to effectively solve the lack of low-temperature cracking resistance of epoxy resins because its unique reactive end groups can react with epoxy resin reactive groups to produce flexible chain blocks, forming chemical bonding interactions and obtaining block copolymers [20,21], which can achieve toughening.

In summary, the objective of this study is to explore the road performance of terminated carboxylated nitrile butadiene rubber (CTBN) epoxy-modified asphalt and to explore the feasibility of terminated carboxylate nitrile butadiene rubber epoxy asphalt as a permeable pavement in cold regions. To realize this, in this study, CTBN-modified epoxy resin was added to asphalt to make modified asphalt, and the toughness, viscosity, high-temperature stability performance, low-temperature cracking performance, changes in functional groups, and micro-morphology of the modified asphalt, as well as the road performance of asphalt concrete, were investigated [22].

## 2. Experimental Materials and Methods

### 2.1. Materials

**Figure 1:**
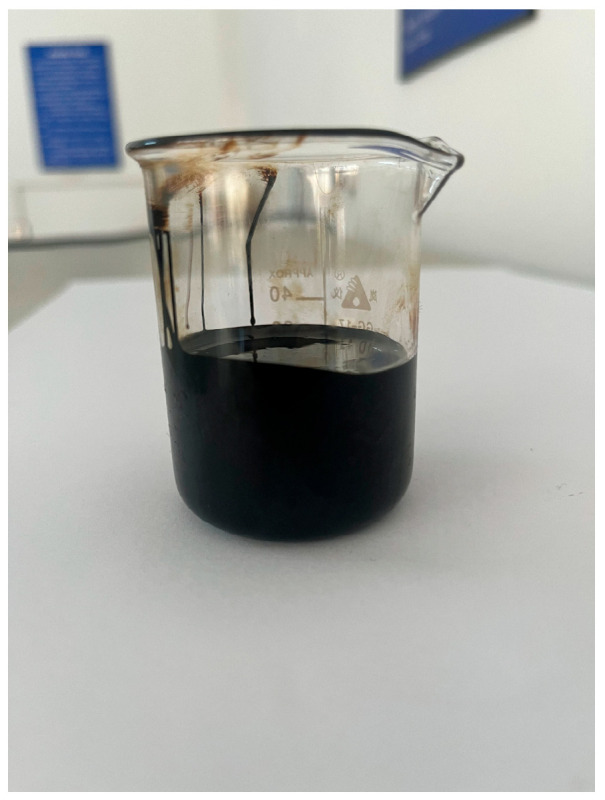
Asphalt.

**Figure 2:**
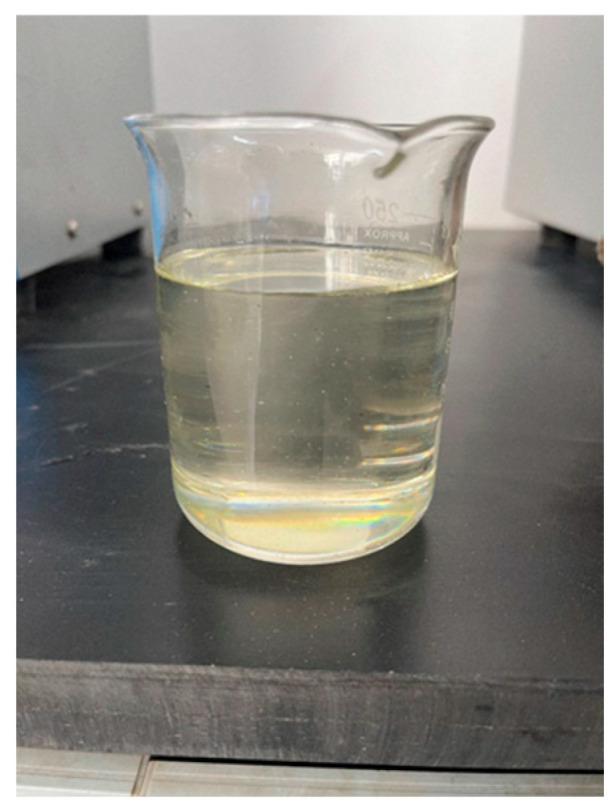
Epoxy resin.

**Figure 3:**
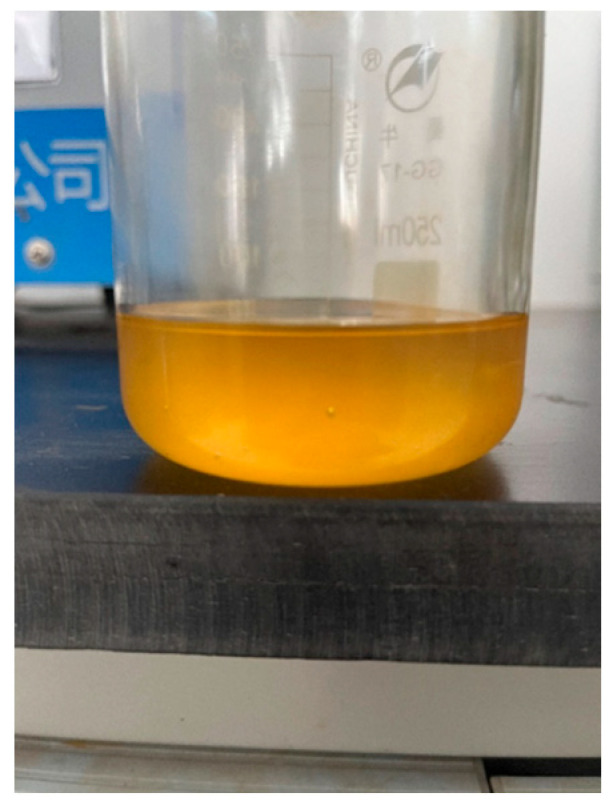
CTBN.

In this study, 90# matrix asphalt, bisphenol A epoxy resin, and liquid-terminated carboxylated nitrile butadiene rubber (CTBN) were selected, as shown in Figure 1, Figure 2 and Figure 3. The technical properties of asphalt, epoxy resin, and CTBN are shown in Table 1 and Table 2. The curing agent of epoxy resin is methyl tetrahydrophthalic anhydride (MTPA), the diluent is C12-14 alkyl glycidyl ether (AGE), and the technical properties of MTPA and AGE are shown in Label 2. The asphalt concrete used in the study is the most common PAC-13 (porous asphalt concrete with a maximum particle size of 13 mm), and the target porosity is 18%. The optimum oil–rock ratio was determined by fly-away and precipitation leakage experiments; basalt was used as coarse and fine aggregates; and limestone mineral powder was used, the technical properties of which are shown in Table 3. In this study, SBS (Styrene-Butadiene-Styrene Block Copolymer) and EP (epoxy resin) were selected as modifiers for porous asphalt mixtures; the gradation curves of the aforementioned concrete mixtures versus the design gradation are presented in Table 4.

### 2.2. Preparation of Modified Bitumen

**Figure 4:**
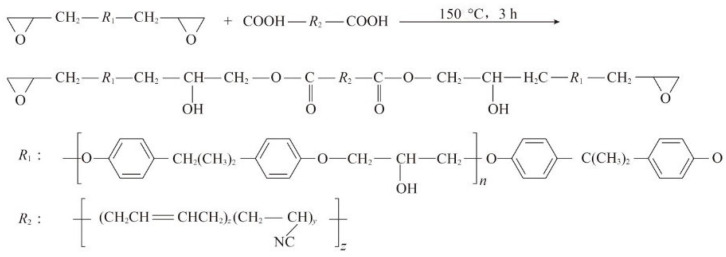
Chemical reaction equations for EP and CTBN.

The epoxy asphalt binding material prepared in this study mainly contains components A and B, of which component A mainly consists of terminal carboxylated nitrile butadiene rubber epoxy resin prepolymer and active diluent, and component B mainly consists of matrix asphalt and curing agent. Terminated carboxylated nitrile butadiene rubber (CTBN) itself is a kind of remote claw polymer; its molecular ends have active functional group carboxyl, which, when mixed with EP, will generate different products according to different conditions (according to the temperature, reaction time is divided into physical modification and chemical modification). This study adopts the chemical modification shown in Figure 4; CTBN and EP were put into a 120 °C environment for 1 h to remove most of the air bubbles and increase its fluidity. Then, both of them were put into the high-speed shear, stirred in the environment of 150 °C for 1 h, and kept at a constant temperature of 150 °C for 2 h to obtain the terminal carboxylated nitrile-butadiene rubber epoxy resin prepolymer, which was then placed in standby at 60 °C after the reaction. The curing agent was preheated to 80 °C, the matrix asphalt was preheated to 125 °C, and the epoxy resin and diluent were preheated to 60 °C. The curing agent and matrix asphalt were mixed with high-speed shear at 120 °C for 30 min to make the B component. The prepolymer and the diluent were stirred for 3 min to make the A component. Finally, the two groups of A and B were put into a high-speed shear machine and sheared at 120 °C for 5 min to obtain the final terminated carboxylated nitrile butadiene rubber epoxy asphalt. The mixture was then cured in an oven at 120 °C for 4 h. Through laboratory pre-tests, the 4-in-1 dosages of CTBN in the asphalt were determined to be 1%, 2%, 3%, and 4% of the epoxy resin, respectively.

The porous asphalt mix in this study was prepared by mixing the CTBN epoxy asphalt, coarse aggregate, fine aggregate, and mineral powder. Due to the unique characteristics of the epoxy resin, which requires self-curing, the preparation method differs from that of ordinary porous asphalt concrete. First, the aggregates (coarse aggregate, fine aggregate, and mineral powder) were placed in a high-temperature environment to evaporate the moisture and then kept in reserve. Meanwhile, the materials for preparing the CTBN epoxy asphalt binder were stored at a specified temperature. Next, the dewatered aggregates were placed in a 120 °C environment and set aside. After preparation, the coarse and fine aggregates were added to the mixer at 120 °C and mixed for 1 min. The CTBN epoxy asphalt binder (excluding the final curing step at 120 °C for 4 h) was then poured into the mixer and mixed for another 1 min. Subsequently, the mineral powder was added to the mixer at 120 °C and mixed for an additional 1 min to obtain the uncured CTBN epoxy porous asphalt concrete. Finally, the uncured CTBN epoxy porous asphalt concrete was poured into designated experimental molds to set its shape and then cured at 120 °C for 4 h to obtain the final CTBN epoxy porous asphalt concrete.

### 2.3. Asphalt Test Methods

1. Tensile Test
  The cured modified bitumen was cooled and demolded under standard test conditions (23 °C ± 2 °C) and subsequently maintained at the same temperature for 2 h to ensure uniform conditioning. The tensile test was performed using a universal material testing machine (Shanghai Tuofeng Instruments Technology Co., Ltd, Shanghai, China) with a tensile rate of 500 mm/min ± 50 mm/min. This test was conducted to evaluate the tensile strength and elongation properties of the modified bitumen, providing insights into its mechanical performance under stress.
2. Brinell Rotational Viscosity Test
  To assess the viscosity characteristics of the modified asphalt during construction, the Brinell rotational viscosity test was conducted using a 27-rotor spindle (Shanghai Changji Geological Instruments Co., Ltd, Shanghai, China) at a rotational speed of 100 RPM. The test was performed at a temperature of 120 °C, with the torque range maintained between 10% and 98%. The objective was to determine the target viscosity and the time required to achieve it, which are critical parameters for evaluating the workability and application performance of the modified asphalt.
3. Temperature Scanning Test
  The temperature scanning experiment was performed using Anton Paar’s MCR rheometer (Anton Paar GmbH, Graz, Austria) to analyze the rheological properties of the asphalt binder. The scanning temperature ranged from 40 °C to 82 °C (increments of 6 °C: 40, 46, 52, 58, 64, 70, 76, and 82 °C) at a constant angular velocity of 10 rad/s. This test aimed to measure the high-temperature rutting resistance of the asphalt binder, providing data on its stiffness and deformation behavior under varying thermal conditions.
4. Bending Beam Rheometer (BBR) Test
  The BBR test was conducted to evaluate the low-temperature cracking resistance of the modified asphalt. Three test temperatures (−12 °C, −18 °C, and −24 °C) were selected to simulate cold climatic conditions. The test measured the creep stiffness (S) and stress relaxation capacity (m) of the asphalt binder, which are critical indicators of its ability to resist thermal cracking and maintain flexibility at low temperatures.
5. Fourier Transform Infrared Spectroscopy (FTIR)
  FTIR analysis was performed to identify the chemical changes and functional group differences between unmodified and CTBN-modified asphalt. The test was conducted over a wavelength range of 400–4000 cm^−1^, capturing the characteristic absorption peaks associated with specific chemical bonds. This analysis provided insights into the chemical modification mechanism and the interaction between CTBN and the asphalt matrix.
6. Atomic Force Microscopy (AFM)
  AFM was employed to characterize the surface morphology of CTBN-modified bitumen at a test temperature of 25 °C. The microscopic images were captured using the tapping mode of AFM (Bruker Corporation, Billerica, MA, USA) with a scanning range of 30 μm × 30 μm. The test results were analyzed using specialized software (NanoScope Analysis 3.0) to study the microstructure and phase distribution of the modified bitumen, offering a detailed understanding of its surface properties and homogeneity.

### 2.4. Asphalt Concrete Test Methods

In this study, unmodified epoxy asphalt with CTBN of 4% of the epoxy resin mass and SBS asphalt porous concrete, which is common in cold regions, were selected for comparison. The asphalt concrete tests were conducted after the porous epoxy asphalt concrete had been set (cured at 120 °C for 4 h):

1. Indoor Rutting Test
  The indoor rutting test was conducted to assess the high-temperature performance of porous asphalt concrete (PAC). The test was performed at a loading rate of 42 cycles per minute, with a tire-applied pressure of 0.7 MPa and a test temperature of 60 °C. The specimens were conditioned at the test temperature for 5 h, followed by an experimental duration of 1 h. This test aimed to measure the rutting depth and deformation resistance of PAC under simulated high-temperature traffic conditions.
2. Water Immersion Marshall Test
  The water immersion Marshall test was carried out to evaluate the water stability of PAC. Two groups of Marshall specimens were prepared, each compacted 50 times. One group was immersed in a 60 °C water bath for 0.5 h, while the other group was immersed for 48 h. After immersion, both groups were maintained at 25 °C, and their Marshall stability values were determined. This test provided insights into the durability and resistance of PAC to water-induced damage.
3. Freeze–Thaw Splitting Test
4. The freeze–thaw splitting test was performed to assess the resistance of PAC to freeze–thaw cycles. The specimens were divided into two groups: one group was tested directly, while the other group underwent a 15-minute water saturation treatment, followed by 0.5 h of static water immersion, 16 h at −18 °C, and 24 h in a 60 °C water bath. Both groups were then placed in a 25 °C water bath for 2 h before testing. The splitting strength was determined at a loading rate of 50 mm/min and a temperature of 25 °C, providing data on the material’s performance under freeze–thaw conditions.
5. Low-Temperature Trabecular Bending Test
  The low-temperature trabecular bending test was conducted to evaluate the low-temperature performance of PAC. The test specimens had dimensions of 250 mm in length, 30 mm in width, and 35 mm in height, with a span of 200 mm. Prior to testing, the specimens were conditioned at −10 °C for 45 min. The test was performed at a loading rate of 50 mm/min to measure the bending strength and flexibility of PAC under low-temperature conditions, simulating its behavior in cold climates.

## 3. Results

### 3.1. CTBN-Modified Asphalt Material Performance Test Results

#### 3.1.1. Tensile Test Results

**Figure 5:**
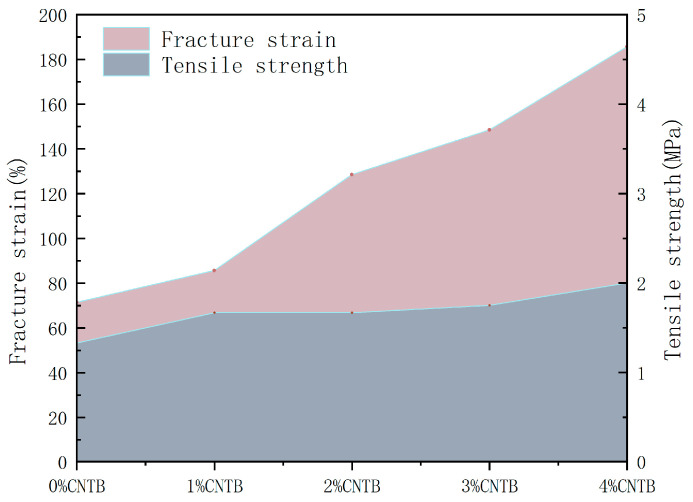
Tensile experiment results.

Figure 5 shows the mechanical properties of CTBN-modified epoxy asphalt. It can be seen that as the CTBN parameter increases, the tensile strength and elongation at the break of the asphalt are also increasing, and in the parameter of 4% it reached the maximum, the tensile strength of 2.03 MPa. The elongation at break reached 185.71%, of which the maximum deviation value is 7.1%. Then, the pure epoxy asphalt tensile strength and elongation at break increased by 66.5% and 260%, respectively. This indicates that the epoxy resin in the epoxy asphalt is compatible with NBR, reaching the homogeneous compatibility state; the flexible molecular chain of CTBN is entangled with the rigid chain segment of the epoxy resin, resulting in the improvement of tensile properties; and the significant increase in elongation at break reflects the significant effect of CTBN on the toughening of epoxy asphalt and improves the brittle condition of the original epoxy asphalt.

#### 3.1.2. Brinell Rotational Viscosity Test Results

**Figure 6:**
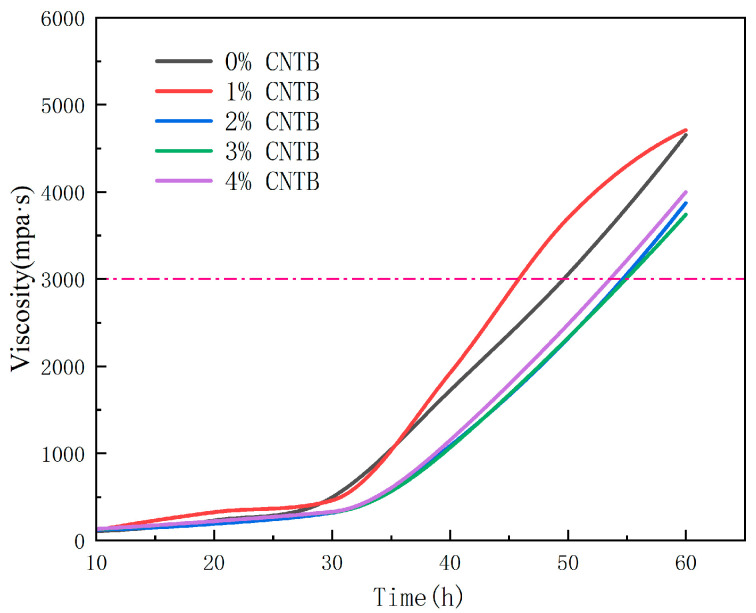
Results of Booker’s rotational viscosity experiment.

Viscosity reflects the viscosity of the asphalt material and represents the frictional and pressure resistance to the flow of asphalt molecules. The viscosity of the asphalt film characterizes the ability to resist deformation at a given shear rate, which greatly affects the preparation of the asphalt mixture. To ensure that the construction phase can be carried out before the modified asphalt has reached a strong degree of curing, the viscosity is strictly controlled within the appropriate range during the mixing, paving, and compaction of the mixture in the field [23]. Figure 6 shows the modified asphalt in different CTBN parameters, where the whole curing rate can be seen; the systems exhibited increased initial viscosity while demonstrating significant curing rate acceleration at 30 min. The curing rate in the CTBN parameter increases in the first increase, then decreases and then increases the phenomenon. The time required to reach 3000 MPa compressive strength was 54 min and 47 min. At 2%, 3%, and 4%, the pre-polymer generated by CTBN and epoxy resin may reduce the curing rate of the epoxy system, while the 1% parameter has a catalytic accelerating effect on the curing system. In summary, the modified asphalt with high parameterization can meet the construction stage with sufficient time.

#### 3.1.3. Bending Beam Rheometer Experiment (BBR) Results

**Figure 7:**
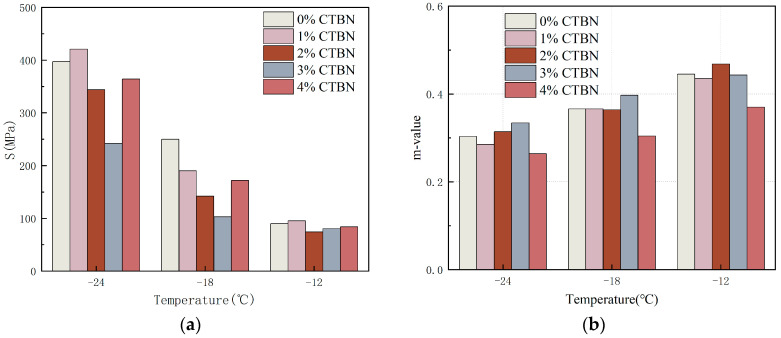
BBR test results for asphalt binder: (**a**) S and (**b**) m values.

If the strength of the asphalt material is too large, it shows brittleness and the pavement is easy to crack and damage; while the larger rate of change of the characterized asphalt strength with time means that the response of the binder still has a certain speed when the temperature drops to make the pavement shrink, which leads to the reduction in tensile stress in the material and the possibility of low-temperature cracking. In the BBR test, lower S values and higher m values indicate better low-temperature cracking performance of asphalt binders [24]. Figure 7 shows the creep strength S and creep rate m of modified epoxy asphalt with different CTBN coefficients. After the addition of CTBN, the S of asphalt shows an increasing and then decreasing trend and reaches the lowest at the 3% coefficient; and the m of asphalt shows an increasing and then decreasing trend, reaching the highest point at 3%. It shows that the addition of CTBN changed the thermal movement ability under low-temperature conditions; the relaxation performance rose, creep deformation ability increased, and it indeed improved the low-temperature cracking resistance of epoxy asphalt, and the effect reached the best when the mass fraction was 3%. The maximum deviation value is 10.1%.

#### 3.1.4. Temperature Scanning Experiment Results

In the temperature scanning experiments, the rutting factor G*/sinδ characterizes the asphalt’s resistance to deformation, and the larger its value, the stronger the resistance to rutting deformation. The rutting factor of asphalt samples shows a decreasing trend with increasing temperature, which is due to the transformation of asphalt from a viscoelastic body to a viscous fluid under the action of high temperature, which reduces the shear deformation resistance and rutting resistance [25].

**Figure 8:**
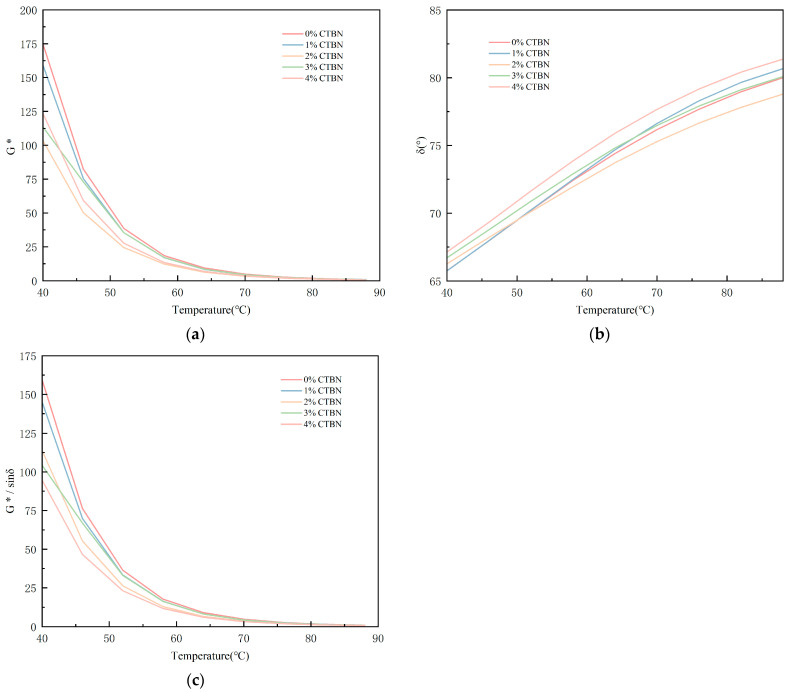
Experimental results of temperature scanning (**a**) complex modulus G*, (**b**) phase angle δ, and (**c**) rutting factor G*/sinδ.

As shown in Figure 8a, the G* of modified epoxy asphalt decreases with increasing temperature, and the rate of decrease also decreases. Increasing the content of CTBN decreases the G* of the modified asphalt, but at a parameter of 4%, the G* increases again. Figure 8b shows that δ increases with increasing temperature, indicating that the heated bitumen loses some of its elasticity and increases the proportion of viscous components. The addition of CTBN increases the δ of the modified asphalt, reaching a maximum of 4%. Figure 8c shows that the rutting factor of epoxy-modified asphalt decreases as the temperature increases, indicating that the higher the temperature, the more prone to rutting the epoxy-modified asphalt is. Under the same environment and temperature, the addition of CTBN decreases the G* and rutting factor of asphalt binder, which shows that the addition of CTBN reduces the high-temperature deformation resistance of asphalt binder to a certain extent, i.e., reduces its high-temperature performance, but the increase in δ also shows that the addition of CTBN increases the elastic component of asphalt binder.

#### 3.1.5. Fourier Transform Infrared Spectroscopy (FITR) Test Results

**Figure 9:**
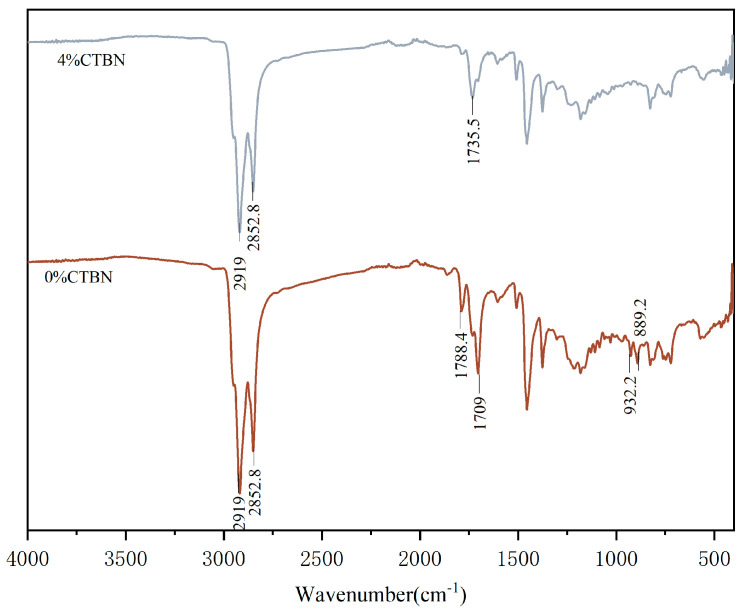
FTIR test results of CTBN-modified epoxy asphalt binder.

Figure 9 shows the comparison of infrared spectral analysis before and after the addition of CTBN-modified epoxy asphalt adhesive, in which the 0% parameter CTBN and 4% parameter CTBN are both for the state after the curing has been completed. From the figure, it can be seen that the two kinds of asphalts contain both symmetric and antisymmetric telescopic vibrational absorption peaks of ${-CH}_{2}$ (2852.8 cm^−1^ and 2919 cm^−1^) [26], the common tube energy group of asphalt. Comparing the two, the disappearance of −COOH absorption peaks at 1709 cm^−1^ and 1788.4 cm^−1^ in 0% CTBN, and the formation of 1735 cm^−1^ −COOR peak in 4% CTBN, indicate that the epoxy group of epoxy resin and the carboxyl group of CTBN complete the reaction to obtain a new functional group [27], which is a chemical reaction, and this proves that the preparation method is correct. The disappearance of the C−H absorption peaks [28] (932.2 cm^−1^ and 889.2 cm^−1^) may be the formation of a cross-linking polymerization network, which also indirectly proves the chemical reaction between the epoxy resin and CTBN.

#### 3.1.6. Atomic Force Microscopy (AFM) Test Results

**Figure 10:**
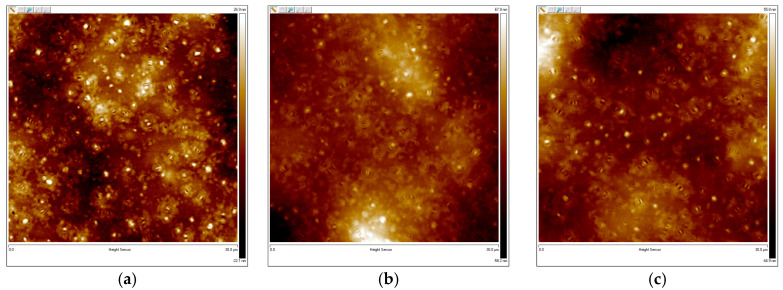
Two-dimensional AFM image (**a**) 0% CTBN, (**b**) 2% CTBN, (**c**) 4% CTBN.

**Figure 11:**
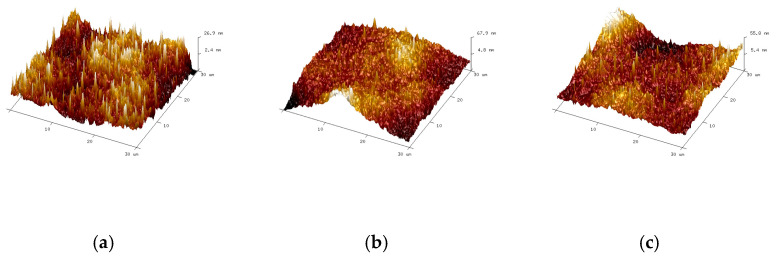
Three-dimensional AFM image (**a**) 0% CTBN, (**b**) 2% CTBN, (**c**) 4% CTBN.

Figure 10 shows the three phases of the AFM results for CTBN-modified epoxy asphalt. As shown in the figure, there is a series of bee-like white and black lines distributed on the darker areas, which are called the “bee phase”, “bee casing phase”, and “gap phase” in the figure. The katana phase is a bee-like structure, the peri phase is the peripheral region of the bee-like structure, and the subphase is the neighboring region of the peri phase [2]. H.L. ZHANG et al. separated asphaltene and maltene into asphaltene and did not find a “bee-like” structure when observing them individually. Therefore, it is believed that the appearance of a “bee-like” structure is caused by the presence of asphaltene in asphalt [29]. The “bee-like” structures are further suggested to be waxy molecules (highly aromatic and long alkyl chain asphaltenes) [30]. The pure epoxy asphalt without added CTBN as shown in Figure 10a has a large bee-like structure with a clear difference between the peripheral and secondary phases and a peri-centralized distribution with many bee-like structures connected. As shown in Figure 10b,c, with the addition of CTBN, the AFM results of the asphalt binder changed greatly, and the original large bee-like structure gradually decreased, the number decreased, the distribution was more dispersed, the difference between the peripheral phase and the subphase gradually decreased, and the overall tendency was more homogeneous. The reason may be pure epoxy asphalt, epoxy resin, and asphalt within the formation of the phase cross-structure; to be cured after the epoxy system, asphalt is cured in epoxy resin to separate, the asphalt matrix is more concentrated in a part of the structure, and the range of motion is reduced, although the two interspersed can improve the rheological properties of the asphalt, also further limiting the connectivity of the asphalt matrix microscopically. This also leads to epoxy asphalt at low temperatures, showing a large strength and more brittle characteristics. CTBN and epoxy resin were added to form a pre-polymer after joining the asphalt system to complete the curing, making the performance of the asphalt matrix distribution more uniform because of the CTBN and the epoxy resin’s mutual solubility and chemical reaction; CTBN molecules interspersed with the epoxy resin in the main chain change the epoxy resin and the asphalt of the original distribution of structures. This changes the original distribution structure of the epoxy resin and asphalt and increases the free diffusion and movement space of the asphalt matrix, which makes the asphalt matrix and the new substances after the reaction more uniformly distributed. On a macro level, CTBN offers more like a lubrication effect, and its internal lubrication effect makes the flexibility of the modified material increase and the rigidity decrease. Figure 11 shows the 3D results of the AFM of the asphalt binder. It can be seen that in the 3D results, a large number of peaks and valleys are distributed, which form a large number of crystals and surface folds in the class. Moreover, these crystallites are shown as bee-like structures in the 2D results. The same as in the 2D structure, in the pure epoxy asphalt without the addition of CTBN, there are a large number of robust crystalline structures; with the addition of CTBN, the size and number of crystals began to gradually reduce, which indicates that the CTBN changed the crystal structure of epoxy asphalt, enhancing its stability and homogeneity, and at the same time, this also verifies the 2D results.

### 3.2. CTBN-Modified Porous Asphalt Concrete Road Performance Test Results

To evaluate the road performance of CTBN-modified epoxy porous asphalt concrete more objectively, a 4% mass fraction of SBS-modified asphalt was added as a comparison in this study, and epoxy resin (EP), CTBN-modified epoxy asphalt with 4% mass fraction, and SBS-modified asphalt were selected.

#### 3.2.1. High-Temperature Properties of Porous Asphalt Concrete

Dynamic stability (defined as 1 mm rut depth caused by wheel loading cycles on the specimen at 60 °C) was used to characterize the high-temperature rutting stability of asphalt concrete in wheel loading tests, with higher dynamic stability indicating better rutting stability [31]. As shown in Table 5, the stability of SPAC, EPAC, and CEPAC concrete is 2550.6, 14,318.2, and 18,529.4 times/mm at 60 °C. The maximum deviation value is 5.5%. The dynamic stability of EPAC and CEPAC is much higher than that of SPAC, and the dynamic stability of CEPAC is improved by 29.4% compared to that of EPAC. The high-temperature performance of CEPAC compared to that of EPAC shows no significant decrease, and there is a partial enhancement, which may be because the addition of CTBN increases its partial viscosity and homogeneity, increases the adhesion with the mineral material, and improves the high-temperature stability of the asphalt concrete so that side by side, the modified epoxy asphalt has a higher heat resistance than the pure epoxy asphalt.

#### 3.2.2. Low-Temperature Properties of Porous Asphalt Concrete

**Figure 12:**
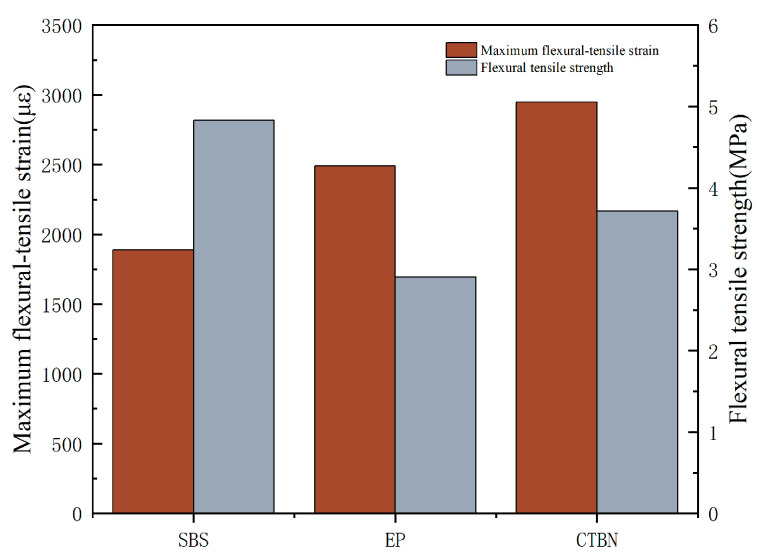
Low-temperature trabecular bending test results.

Figure 12 shows the flexural tensile strength and stiffness modulus of three different PACs at −10 °C. The maximum flexural tensile strains of SPAC, EPAC, and CEPAC are 2816.688, 1695.456, and 2165.856, respectively, and the flexural tensile strengths of EPAC and CEPAC are indeed much higher compared to that of SPAC, which has a stronger resistance to low-temperature deformation. The maximum deviation value is 8.1%. Compared with EPAC, the flexural tensile strength of CEPAC is 5.53 MPa, which is 18.3% larger than that of EPAC. With the increase in CTBN doping, the flexural tensile strain is improved, the low-temperature performance is improved, and the durability is strengthened, so that the allowable strain maximum value is increased in the process of low-temperature cracking. With the increase in CTBN dosage, the flexural tensile strain is improved, the durability is strengthened, the allowable strain is increased in the process of low-temperature cracking, the low-temperature brittleness of epoxy asphalt is improved to a certain extent, and the flexural tensile strain is improved by about 27.74%.

#### 3.2.3. Water Stability Properties of Porous Asphalt Concrete

**Figure 13:**
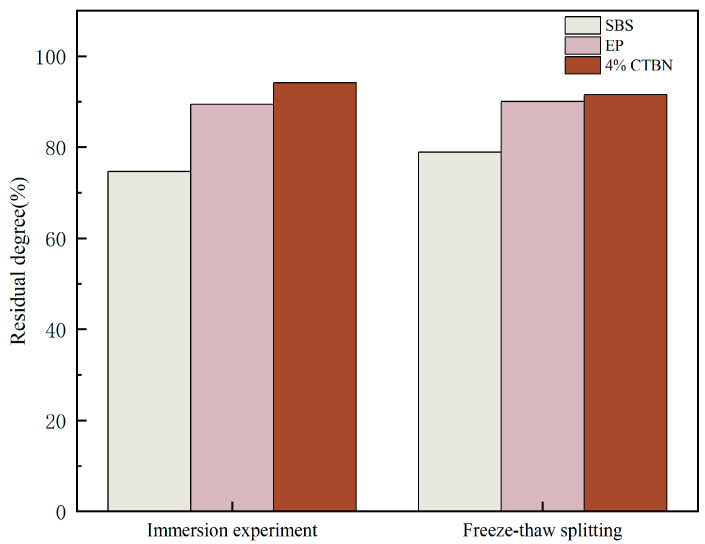
Water stability test results.

The nature of water damage to asphalt concrete is due to the lack of adhesion between the asphalt and aggregate as well as the lack of cohesion within the asphalt [32]. Figure 13 shows the residual stability and freeze–thaw split strength ratio of three kinds of PACs in the water-immersion Marshall test and freeze–thaw split test; with the addition of CTBN, CEPAC has the largest residual stability and freeze–thaw split strength ratio, which reach 94.1% and 94.5%. The maximum deviation value is 4.2%. CTBN and EP form a stable “island structure” under the action of a curing agent. Asphalt as a dispersed phase is distributed in the “island structure”, which improves the cohesion of epoxy asphalt; makes its combination with the aggregate more solid; makes it better adhere to the aggregate, hindering the asphalt peeling caused by the decrease in cohesion in the process of water damage; and improves the sensitivity of epoxy asphalt to water.

## 4. Conclusions

This study proposes the application of permeable pavement technology to address the overlay effect in cold regions, utilizing terminal carboxylated nitrile rubber (CTBN)-modified epoxy resin as a pavement material modifier. The performance of CTBN-modified epoxy asphalt and its corresponding asphalt concrete under varying parameters was systematically investigated. Furthermore, the mechanism of CTBN in asphalt was thoroughly elucidated. Based on the research findings, the following key conclusions were drawn:

1. CTBN significantly enhances the tensile strength and toughness of epoxy asphalt without accelerating the curing time of the epoxy system. While it slightly reduces the high-temperature rutting resistance, it improves the material’s elasticity. Additionally, CTBN effectively enhances the low-temperature cracking resistance of epoxy asphalt, as evidenced by a reduction in the low-temperature stiffness modulus (S) and an improvement in the stress relaxation capacity (m).
2. CTBN-modified epoxy asphalt undergoes chemical modification, wherein the reaction between CTBN and epoxy resin generates new functional groups. This alters the overall distribution of the epoxy asphalt, increasing its fluidity and homogeneity, thereby providing a theoretical basis for the improvement in macroscopic performance.
3. Compared to EPAC and SPAC, CEPAC exhibits superior high-temperature performance, low-temperature performance, and water stability due to the formation of a stable “sea-island structure”. Although its low-temperature tensile strain is slightly inferior to that of SPAC, CEPAC is more suitable as a pavement material for permeable pavements in cold regions when considering overall performance.

The results of this study demonstrate that CTBN-modified epoxy asphalt can largely meet the engineering requirements for permeable pavements in cold regions, offering a viable option for material selection. However, to adapt to the frequent thermal fluctuations in cold climates, further in-depth research is necessary, particularly to explore the performance of CTBN-modified epoxy asphalt and CEPAC under freeze–thaw cycle conditions. Future studies should focus on optimizing material formulations and construction techniques to enhance durability and stability under extreme climatic conditions, thereby promoting the widespread application of permeable pavement technology in cold regions.

## Figures and Tables

**Table 1:** Pure asphalt binder and epoxy resin technical indicators.

Material	Technical Indicators	Value
90# Matrix Asphalt Bonding Agent	Needle penetration (25 °C, 100 g, 5 s)/0.1 mm	89
	Softening point (TR&B)/°C	51
	Elongation (15 °C, 5 cm/min)/cm	121
	Dynamic viscosity (60 °C)/Pa·s	199
epoxy resin	Epoxy equivalent/(g/mol)	185
	Inorganic chlorine value/(mg/kg)	7

**Table 2:** Technical specifications of curing agents and diluents.

Material	Technical Indicators	Value
Methyl tetrahydrophthalic anhydride	Viscosity (25 °C)/mPa⋅s	41
	Anhydride base content/%	42.3
	Free acid/%	0.1
C12-14 alkyl glycidyl ethers	Viscosity (25 °C)/mPa⋅s	8.4
	Epoxy equivalent/(ep/100 g)	0.33
	Hydrolyzed oxygen/ppm	370

**Table 3:** Mineral aggregate specifications.

Material	Coarse Aggregate	Fine Aggregate	Mineral Powder
Relative apparent density/(g/cm^3^)	2.821	2.740	2.685
Crushing value/%	10.5	/	/
Water absorption/%	0.4	1.0	/
Needle flake content/%	5.9	14.7	/

**Table 4:** Grading design of PAC.

Mixture	Passing (by Mass) Under Different Sieve Sizes (mm)%										Binder Content/%	Void Ratio/%
	16	13.2	9.5	4.75	2.36	1.18	0.6	0.3	0.15	0.075		
SBS	100.0	95.0	69.0	21.0	19.2	14.5	10.0	6.80	5.2	4.5	5.0	18.2
EP (EP + CTBN)	100.0	95.0	69.0	21.0	19.2	14.5	10.0	6.80	5.2	4.5	5.0	18.3

**Table 5:** Different asphalt OGFC-13 dynamic stabilities.

Type of Asphalt	Displacement (mm)		Dynamic Stability (Times/mm)
	45 min	60 min	
SPAC	12.29	12.54	2550.6
EPAC	1.07	1.11	14,318.2
CEPAC	0.49	0.525	18,529.4

## Data Availability

The original contributions presented in the study are included in the article, further inquiries can be directed to the corresponding authors.
